# Controlling the Overfitting of Heritability in Genomic Selection through Cross Validation

**DOI:** 10.1038/s41598-017-14070-z

**Published:** 2017-10-20

**Authors:** Zhenyu Jia

**Affiliations:** Department of Botany and Plant Sciences, University of California, Riverside, USA

## Abstract

In genomic selection (GS), all the markers across the entire genome are used to conduct marker-assisted selection such that each quantitative trait locus of complex trait is in linkage disequilibrium with at least one marker. Although GS improves estimated breeding values and genetic gain, in most GS models genetic variance is estimated from training samples with many trait-irrelevant markers, which leads to severe overfitting in the calculation of trait heritability. In this study, we demonstrated overfitting heritability due to the inclusion of trait-irrelevant markers using a series of simulations, and such overfitting can be effectively controlled by cross validation experiment. In the proposed method, the genetic variance is simply the variance of the genetic values predicted through cross validation, the residual variance is the variance of the differences between the observed phenotypic values and the predicted genetic values, and these two resultant variance components are used for calculating the unbiased heritability. We also demonstrated that the heritability calculated through cross validation is equivalent to trait predictability, which objectively reflects the applicability of the GS models. The proposed method can be implemented with the Mixed Procedure in SAS or with our R package “GSMX” which is publically available at https://cran.r-project.org/web/packages/GSMX/index.html.

## Introduction

Plant breeding is to produce desired characteristics by changing the traits of plants. Traditionally, we directly selected plants with desirable characteristics for propagation. In the past two decades, molecular techniques have been used for indirectly selection^1–5^, for example, marker assisted selection (MAS). Markers (e.g., DNA/RNA variations), which are in linkage disequilibrium (LD) with quantitative trait loci (QTL), are used for indirect selection of genetic determinants of traits of interest. Linear regression models can be used to assess the association between the traits and the markers using training sample in which the phenotypes and genotypes are known for each subject^6,7^. Phenotypic values are regressed on the genotypic values of markers, and statistical hypothesis test is performed on each marker. Criterion, such as threshold in *p* value or in the logarithm of the odds ratio (LOD score), are used for detection of markers/QTL with significant effects^8^. The selected markers are used to form a linear genetic model (*e*.*g*., least squares model) which will be used for the recurrent selection process. Two criterions are commonly used for evaluating the performance of the genetic models built from training samples, *i*.*e*., heritability and predictability^9,10^. The heritability is defined as the proportion of variance that can be explained by the genetic model, which is equivalent to the R^2^ in linear regression analysis. The heritability for the training sample can be conveniently calculated with analysis of variance (ANOVA). Whereas, the predictability for the training sample is defined as the squared correlation between the observed phenotypes and the predicted phenotypes that are calculated using the genetic model under consideration. If the size of the training sample is larger than the number of the markers (a requirement for regular linear model), the heritability and predictability are actually measuring the same quantity. Note that the heritability and the predictability associated with a genetic model only reflect the genetic structure for the population from which the training sample has been drawn; much lower levels of heritability and predictability may be obtained if this genetic model (developed from training sample) is applied to a sample which comes from a distinct population, for example, a population with very different genetic background.

With the emergence of the low-cost but high-throughput sequencing technologies, we can easily increase the number of markers and their density to enjoy increased resolution of QTL mapping^11–13^. In many MAS or QTL mapping studies, the number of markers is much larger than the number of individuals, *i*.*e*., *p* ≫ *n*, where *p* is the number of markers (or parameters in the regression models) and *n* is the sample size. Under this circumstance, the least squares estimation does not work; likewise, ANOVA cannot be directly applied to the data. Rather, sophisticated strategies should be used to reduce the dimensionality of the analysis. To fit ANOVA, one can first reorganize the data by categorizing subjects in the sample into groups based on their genotypes (for example, recombinant inbred lines (RILs)), and then analyze the variances between these groups (or lines). Note we used ‘reorganization of data’ to represent rearrangement of the data using a newly defined independent/predictive variable, *i*.*e*., we used the RILs (groups defined based on genotypes) as an independent variable for the ANOVA analysis. Nevertheless, samples with similar genotypes may be placed into different groups, yielding too many groups (or parameters) for the model and therefore overfitting the data. This is similar to the situation where too many ‘leaves’ are used to overly fit a ‘tree’ classification model. Therefore, the heritability calculated using this strategy through ANOVA is likely to be overestimated. Alternatively, variable selection strategies can be used in regression models to substantially reduce the number of parameters by assuming that most markers are irrelevant^14–17^. However, it has been commonly accepted that quantitative traits are determined by many genes including some major genes as well as a large number of genes with small effects. Major QTL only explain part of the total variation; on the other hand, significant portion of variation is attributed to many QTL with small or even tiny effects across genome which usually do not survive the statistical selection criterion. ‘Complete modeling’ by considering all QTL on the genome including those with small effects has potential to improve the performance of the genetic models. It is worthy of noting here that the heritability calculated through ANOVA using reorganized data is no longer equivalent to the predictability calculated using regression models since ANOVA analyzes the parameters that are derived from the genotypes rather than genotypes themselves. However, correlations are expected between the heritability from ANOVA (with rearranged data) and the predictability from regression models.

Genomic selection (GS) provides solutions to ‘complete modelling’. GS is a form of MAS in which genetic markers covering the whole genome are used so that all QTL are in linkage disequilibrium with at least one marker^9,10,16,18,19^. Models including random effects (*e*.*g*., mixed models) are used to reduce the dimensionality of the analysis. In GS, the effects of all markers, including the markers with major effects as well as many more markers with small effects, are first estimated from training sample, and then are used to form a genetic model for predicting genetic values for unphenotyped individuals. It has been indicated that GS models are more predictive and potentially more effective than classical MAS schemes or use of pedigree; therefore, the studies on GS become more and more popular. However, in GS analyses, the whole-genome markers are used to fit the regression model and estimate the covariance (kinship) between individuals in the training set; such information is subsequently used to calculate the parameters including variance components which are used to calculate trait heritability. The majority of the loci on the genome are neutral to the trait of interest; only *causative loci* (small portion of the genome) are contributing to the variation of the trait. Including the large number of the neutral loci in the regression model likely overfits the data. In the current study, we used intensive simulated studies to demonstrate that the genetic variance is overestimated using regular GS settings and trait heritability is thereafter exaggerated. In order to realistically reflect the applicability of the GS models that are developed using training samples, cross validation should be used to control such overfitting. In the study, we proposed a simple algorithm to calculate unexaggerated trait heritability in GS analysis. Our new method echoes the previous studies where cross validation has been used to reduce the bias of estimation of the predictability (or prediction accuracy) due to the environmental sampling error, genotypic sampling error, or both^20,21^. Compared with the previous efforts, our focus is to provide an effective control on the overfitting of heritability incurred by the excessive non-relevant markers included in the GS analysis, which has been overlooked in the literature. In the new method, a simple solution was proposed to estimate genetic covariance and eventually trait heritability using the variance of the genetic values predicted through cross validation. Hence, the aims of the study include (1) proof of the overfitting due to the inclusion of a large number of trait-irrelevant loci in the GS analyses and the similar overfitting in ANOVA approaches, (2) demonstration of effective control of such overfitting by the new method, and (3) showing that the heritability is equivalent to the predictability (or accuracy of prediction) when such overfitting is controlled. The proposed new method provides an accurate estimation of trait heritability or trait predictability, and objectively reflects the applicability of the GS models when they are applied to independent populations. The algorithms can be conveniently implemented using the Mixed Procedure in SAS or can be implemented using a newly developed R package “GSMX” (https://cran.r-project.org/web/packages/GSMX/index.html). The proposed method in this study has been demonstrated by a series of Monte Carlo simulation experiments and a real data analysis in rice.

## Materials and Methods

### Mixed Model

Mixed model is a specially designed method to include fixed and random effects in a single regression model. Fixed effects represent factors that experimenters directly manipulate and are often repeatable, whereas random effects represent the outcome due to random selection of a sample from the entire population (sources of random variation)^20^. Mixed model is commonly described using the following regression model

1 $${\bf{y}}={\bf{X}}{\boldsymbol{\beta }}+{\bf{Z}}{\boldsymbol{\gamma }}+{\boldsymbol{\varepsilon }},$$

where **y** is the observed response (univariate) of *n* individuals, **X** is a *n* × *q* design matrix for the fixed effects **β** (*q* × 1 vector), **Z** is an *n* × *p* design matrix for the random effects **γ** (*p* × 1 vector), and **ε** is an *n* × 1 vector of random errors. The random effects are assumed to be independently and normally distributed, as indicated by ${\boldsymbol{\gamma }} \sim {\rm{N}}(0{\boldsymbol{,}}{\bf{I}}{\sigma }_{\gamma }^{2})$. The residual errors are also normally distributed ${\boldsymbol{\varepsilon }} \sim {\rm{N}}(0{\boldsymbol{,}}{\bf{I}}{\sigma }^{2})$. The expectation of **y** is ${\rm{E}}({\bf{y}})={\bf{X}}{\boldsymbol{\beta }}$ and the variance-covariance matrix of **y** is

2 $${\rm{Var}}({\bf{y}})={\bf{V}}={\bf{Z}}{{\bf{Z}}}^{T}{\sigma }_{\gamma }^{2}+{\bf{I}}{\sigma }^{2}$$

The variance components, $\theta =\{{\sigma }_{\gamma }^{2},{\sigma }^{2}\}$, can be estimated using the restricted maximum likelihood (REML) method whose log likelihood function is defined by

3 $${\rm{L}}(\theta )=-\frac{1}{2}\,\mathrm{ln}|{\bf{V}}|-\frac{1}{2}\,\mathrm{ln}|{{\bf{X}}}^{T}{{\bf{V}}}^{-1}{\bf{X}}|-\frac{1}{2}{({\bf{y}}-{\bf{X}}\hat{{\boldsymbol{\beta }}})}^{T}{{\bf{V}}}^{-1}({\bf{y}}-{\bf{X}}\hat{{\boldsymbol{\beta }}}),$$

where $\hat{{\boldsymbol{\beta }}}={({{\bf{X}}}^{T}{{\bf{V}}}^{-1}{\bf{X}})}^{-1}({{\bf{X}}}^{T}{{\bf{V}}}^{-1}{\bf{y}})$. Alternatively, the maximum likelihood (ML) method can be used to estimate the parameters,

4 $${\rm{L}}(\theta )=-\frac{1}{2}\,\mathrm{ln}|{\bf{V}}|-\frac{1}{2}{({\bf{y}}-{\bf{X}}\hat{{\boldsymbol{\beta }}})}^{T}{{\bf{V}}}^{-1}({\bf{y}}-{\bf{X}}\hat{{\boldsymbol{\beta }}}).$$

Numerous algorithms can be used to maximize the above likelihood functions and get the REML or ML estimates of the parameters.

### The Best Linear Unbiased Prediction (BLUP)

Various approaches (*e*.*g*., G-BLUP^23^, BayesB^10^, and MIXTURE^24^) may be used for estimation of the genetic effects and development of a prediction model; however, BLUP based method generally gave the higher prediction accuracy due to the fact that a complex trait is usually explained by many genes^21^. In the study, we mainly focus on the BLUP approach on the basis of mixed model. In the general situation, if covariance among genomic loci and covariance of residual errors are considered (in contrast to the independent random effects and independent residual errors described in Eq. (2)), we assume the random effects follow a normal distribution as denoted by ${\boldsymbol{\gamma }} \sim {\rm{N}}({\bf{0}},{\bf{G}}{\sigma }_{\gamma }^{2})$ and the residual errors follow a normal distribution as denoted by ${\boldsymbol{\varepsilon }} \sim {\rm{N}}({\bf{0}},{\bf{R}}{\sigma }^{2})$, where **G** is a variance-covariance structure for the random effects and **R** is a variance-covariance structure for the residual errors. In this case, the variance of the model becomes

5 $${\rm{Var}}({\bf{y}})={\bf{V}}={\bf{Z}}{\bf{G}}{{\bf{Z}}}^{T}{\sigma }_{\gamma }^{2}+{\bf{R}}{\sigma }^{2}$$

The random and fixed effects are estimated from Henderson’s mixed model Eq. 21,

6 $$[\begin{array}{cc}{{\bf{X}}}^{T}{{\bf{R}}}^{-1}{\bf{X}} & {{\bf{X}}}^{T}{{\bf{R}}}^{-1}{\bf{Z}}\\ {{\bf{Z}}}^{T}{{\bf{R}}}^{-1}{\bf{X}} & {{\bf{Z}}}^{T}{{\bf{R}}}^{-1}{\bf{Z}}+{{\bf{G}}}^{-1}/\lambda \end{array}]\,[\begin{array}{c}{\boldsymbol{\beta }}\\ {\boldsymbol{\gamma }}\end{array}]=[\begin{array}{c}{{\bf{X}}}^{T}{{\bf{R}}}^{-1}{\bf{y}}\\ {{\bf{Z}}}^{T}{{\bf{R}}}^{-1}{\bf{y}}\end{array}],$$

where $\lambda ={\sigma }_{\gamma }^{2}/{\sigma }^{2}$. The BLUE (best linear unbiased estimation) and BLUP (best linear unbiased prediction) of the fixed effects and random effects are obtained via

7 $$[\begin{array}{c}\hat{{\boldsymbol{\beta }}}\\ \hat{{\boldsymbol{\gamma }}}\end{array}]={[\begin{array}{cc}{{\bf{X}}}^{T}{{\bf{R}}}^{-1}{\bf{X}} & {{\bf{X}}}^{T}{{\bf{R}}}^{-1}{\bf{Z}}\\ {{\bf{Z}}}^{T}{{\bf{R}}}^{-1}{\bf{X}} & {{\bf{Z}}}^{T}{{\bf{R}}}^{-1}{\bf{Z}}+{{\bf{G}}}^{-1}/\lambda \end{array}]}^{-1}[\begin{array}{c}{{\bf{X}}}^{T}{{\bf{R}}}^{-1}{\bf{y}}\\ {{\bf{Z}}}^{T}{{\bf{R}}}^{-1}{\bf{y}}\end{array}].$$

The variance-covariance matrix of the BLUE and BLUP is

8 $${\rm{Var}}\,[\begin{array}{c}\hat{{\boldsymbol{\beta }}}\\ \hat{{\boldsymbol{\gamma }}}\end{array}]={[\begin{array}{cc}{{\bf{X}}}^{T}{{\bf{R}}}^{-1}{\bf{X}} & {{\bf{X}}}^{T}{{\bf{R}}}^{-1}{\bf{Z}}\\ {{\bf{Z}}}^{T}{{\bf{R}}}^{-1}{\bf{X}} & {{\bf{Z}}}^{T}{{\bf{R}}}^{-1}{\bf{Z}}+{{\bf{G}}}^{-1}/\lambda \end{array}]}^{-1}{\sigma }^{2}.$$

### Genomic Selection Model

We adopted the mixed model to describe the variation of phenotypic values in genomic selection, where the fixed effects usually represent controllable and repeatable factors, such as age, location, treatment etc., and the random effects represent the genetic effects for the loci/markers on the genomes. The effects of most loci are close to zero; thus, the normal distribution of random effects are suitable to model the behavior of the genetic effects.

In genomic selection, we rewrite Eq. (1) as the following linear model,

9 $${\bf{y}}={\bf{X}}{\boldsymbol{\beta }}+\sum _{k=1}^{m}{{\bf{Z}}}_{k}{\gamma }_{k}+{\boldsymbol{\varepsilon }},$$

where ${{\bf{Z}}}_{k}$ is a column vector of genotype indicators for marker *k* and ${\gamma }_{k}$ is the marker effect. The genotypic value of marker *k* for individual *j* in a F2 population is defined as

10 $${Z}_{jk}=\{\begin{array}{cc}\begin{array}{c}1\\ 0\\ -1\end{array} & \begin{array}{c}{{\rm{for}}{\rm{A}}}_{1}{{\rm{A}}}_{1}\\ {{\rm{for}}{\rm{A}}}_{1}{{\rm{A}}}_{2}\\ {{\rm{for}}{\rm{A}}}_{2}{{\rm{A}}}_{2}\end{array}\end{array},$$

where $j=1\ldots n$, A_1_ is the reference allele, and A_2_ is minor allele. We assume that ${\gamma }_{k} \sim {\rm{N}}(0,{\sigma }_{\gamma }^{2})$ for all *p* = 1$,\,\ldots ,\,m$, and ${\boldsymbol{\varepsilon }} \sim {\rm{N}}({\bf{0}},{\bf{I}}{\sigma }^{2})$ so that

11 $${\rm{Var}}({\bf{y}})=\sum _{k=1}^{m}{{\bf{Z}}}_{k}{{\bf{Z}}}_{k}^{T}{\sigma }_{\gamma }^{2}+{\bf{I}}{\sigma }^{2}=\frac{1}{m}\sum _{k=1}^{m}{{\bf{Z}}}_{k}{{\bf{Z}}}_{k}^{T}(m{\sigma }_{\gamma }^{2})+{\bf{I}}{\sigma }^{2}={\bf{K}}\text{'}{\sigma }_{A}^{2}+{\bf{I}}{\sigma }^{2},$$

where

12 $${\bf{K}}\text{'}=\frac{1}{m}\sum _{k=1}^{m}{{\bf{Z}}}_{k}{{\bf{Z}}}_{k}^{T}$$

is a marker-inferred kinship matrix^22^ and

13 $${\sigma }_{A}^{2}=m{\sigma }_{\gamma }^{2}$$

is called the polygenic variance. Let us define ${\boldsymbol{\xi }}=\sum _{k=1}^{m}{{\bf{Z}}}_{k}{\gamma }_{k}$ as the polygene and rewrite the mixed model (9) using

14 $${\bf{y}}={\bf{X}}{\boldsymbol{\beta }}+{\boldsymbol{\xi }}+{\boldsymbol{\varepsilon }}{\rm{.}}$$

The variance of **y** is

15 $${\rm{Var}}({\bf{y}})={\rm{Var}}({\boldsymbol{\xi }})+{\rm{Var}}({\boldsymbol{\varepsilon }})={\bf{K}}{\sigma }_{A}^{2}+{\bf{I}}{\sigma }^{2},$$

where **K** is rescaled version of ${\bf{K}}\text{'}$ in order to make sure that ${\sigma }_{A}^{2}$ is comparable with *σ* ^2^, *i*.*e*.,

16 $${\bf{K}}=\frac{{\bf{K}}\text{'}}{{\rm{tr}}({\bf{K}}\text{'})/n}=\frac{n{\bf{K}}\text{'}}{{\rm{tr}}({\bf{K}}\text{'})}.$$

Note that the estimation of this relationship matrix (or kinship matrix) **K** is central to the BLUP based GS analyses (GBLUP). In genetics, the heritability of the trait under study is defined as

17 $${h}^{2}=\frac{{\sigma }_{A}^{2}}{{\sigma }_{A}^{2}+{\sigma }^{2}}.$$

The Henderson’s mixed model Eq. (6) becomes

18 $$[\begin{array}{cc}{{\bf{X}}}^{T}{\bf{X}} & {{\bf{X}}}^{T}\\ {\bf{X}} & {\bf{I}}+{{\bf{K}}}^{-1}/\lambda \end{array}]\,[\begin{array}{c}{\boldsymbol{\beta }}\\ {\boldsymbol{\xi }}\end{array}]=[\begin{array}{c}{{\bf{X}}}^{T}{\bf{y}}\\ {\bf{y}}\end{array}]$$

in genomic selection, where $\lambda =\frac{{\sigma }_{A}^{2}}{{\sigma }^{2}}$. The BLUE and BLUP of the fixed effects and polygenic effect are obtained via

19 $$[\begin{array}{c}\hat{{\boldsymbol{\beta }}}\\ \hat{{\boldsymbol{\xi }}}\end{array}]={[\begin{array}{cc}{{\bf{X}}}^{T}{\bf{X}} & {{\bf{X}}}^{T}\\ {\bf{X}} & {\bf{I}}+{{\bf{K}}}^{-1}/\lambda \end{array}]}^{-1}[\begin{array}{c}{{\bf{X}}}^{T}{\bf{y}}\\ {\bf{y}}\end{array}].$$

The variance-covariance matrix of the BLUE and BLUP is

20 $${\rm{Var}}\,[\begin{array}{c}\hat{{\boldsymbol{\beta }}}\\ \hat{{\boldsymbol{\xi }}}\end{array}]={[\begin{array}{cc}{{\bf{X}}}^{T}{\bf{X}} & {{\bf{X}}}^{T}\\ {\bf{X}} & {\bf{I}}+{{\bf{K}}}^{-1}/\lambda \end{array}]}^{-1}{\sigma }^{2}.$$

### Prediction of Genetic Value

Suppose we hope to utilize the GS model developed as above to predict the genetic values of a new cohort of individuals (for example, in adolescent stage where phenotype of interest has not been fully developed). Let ${{\bf{y}}}_{1}$ be the phenotypic values for the individuals that have been used for developing the GS model and let ${{\bf{y}}}_{2}$ be the individuals for which the phenotypic values or genetic values will be predicted. We rewrite the model (14) as,

21 $${\bf{y}}=[\begin{array}{c}{{\bf{y}}}_{1}\\ {{\bf{y}}}_{2}\end{array}]=[\begin{array}{c}{{\bf{X}}}_{1}{\boldsymbol{\beta }}\\ {{\bf{X}}}_{2}{\boldsymbol{\beta }}\end{array}]+[\begin{array}{c}{{\boldsymbol{\xi }}}_{1}\\ {{\boldsymbol{\xi }}}_{2}\end{array}]+[\begin{array}{c}{{\boldsymbol{\varepsilon }}}_{1}\\ {{\boldsymbol{\varepsilon }}}_{2}\end{array}]$$

The variance-covariance matrix is also partitioned similarly,

22 $${\rm{Var}}({\bf{y}})={\rm{Var}}\,[\begin{array}{c}{{\bf{y}}}_{1}\\ {{\bf{y}}}_{2}\end{array}]=[\begin{array}{cc}{{\bf{V}}}_{11} & {{\bf{V}}}_{12}\\ {{\bf{V}}}_{21} & {{\bf{V}}}_{22}\end{array}]=[\begin{array}{cc}{{\bf{K}}}_{11} & {{\bf{K}}}_{12}\\ {{\bf{K}}}_{21} & {{\bf{K}}}_{22}\end{array}]{\sigma }_{A}^{2}+[\begin{array}{cc}{\bf{I}} & 0\\ 0 & {\bf{I}}\end{array}]{\sigma }^{2}$$

Let ${{\bf{G}}}_{11}={{\bf{K}}}_{11}{\sigma }_{A}^{2}$ and ${{\bf{R}}}_{11}={\bf{I}}{\sigma }^{2}$, then ${{\bf{V}}}_{11}={{\bf{G}}}_{11}+{{\bf{R}}}_{11}$. Other submatrices are similarly defined. We then have

23 $${\rm{Var}}\,[\begin{array}{c}{{\bf{y}}}_{1}\\ {{\bf{y}}}_{2}\end{array}]=[\begin{array}{cc}{{\bf{V}}}_{11} & {{\bf{V}}}_{12}\\ {{\bf{V}}}_{21} & {{\bf{V}}}_{22}\end{array}]=[\begin{array}{cc}{{\bf{G}}}_{11} & {{\bf{G}}}_{12}\\ {{\bf{G}}}_{21} & {{\bf{G}}}_{22}\end{array}]+[\begin{array}{cc}{{\bf{R}}}_{11} & 0\\ 0 & {{\bf{R}}}_{22}\end{array}].$$

To predict the trait values or genetic values in the test sample, we use the conditional expectation of ${{\bf{y}}}_{2}$ given ${{\bf{y}}}_{1}$ (also called BLUP) which is expressed as

24 $$\begin{array}{c}{\hat{{\bf{y}}}}_{2}={\rm{E}}({{\bf{y}}}_{2}|{{\bf{y}}}_{1})={{\bf{X}}}_{2}\hat{{\boldsymbol{\beta }}}+{{\bf{G}}}_{21}{{\bf{V}}}_{11}^{-1}({{\bf{y}}}_{1}-{{\bf{X}}}_{1}\hat{{\boldsymbol{\beta }}})\\ =\,{{\bf{X}}}_{2}\hat{{\boldsymbol{\beta }}}+{{\bf{K}}}_{21}{\hat{\sigma }}_{A}^{2}{({{\bf{K}}}_{11}{\hat{\sigma }}_{A}^{2}+{\bf{I}}{\hat{\sigma }}^{2})}^{-1}({{\bf{y}}}_{1}-{{\bf{X}}}_{1}\hat{{\boldsymbol{\beta }}})\end{array}$$

Information used for the prediction mainly comes from ${{\bf{G}}}_{21}$, the covariance between individuals in the training sample and individuals in the test sample. The model predictability is defined as the squared correlation between the observed and the predicted ${{\boldsymbol{\xi }}}_{2}$, which is

25 $${r}_{{{\boldsymbol{\xi }}}_{2}{\hat{{\boldsymbol{\xi }}}}_{2}}^{2}=\frac{{{\rm{Cov}}}^{2}({{\boldsymbol{\xi }}}_{2},{\hat{{\boldsymbol{\xi }}}}_{2})}{{\rm{Var}}({{\boldsymbol{\xi }}}_{2}){\rm{Var}}({\hat{{\boldsymbol{\xi }}}}_{2})},$$

where

26 $${{\boldsymbol{\xi }}}_{2}={{\bf{y}}}_{2}-{{\bf{X}}}_{2}\hat{{\boldsymbol{\beta }}}\,{\rm{and}}\,{\hat{{\boldsymbol{\xi }}}}_{2}={\hat{{\bf{y}}}}_{2}-{{\bf{X}}}_{2}\hat{{\boldsymbol{\beta }}}$$

with fixed effects being removed.

### Cross Validation

We often name the sample that is used for developing GS model as training set. In literatures, the variance components (${\sigma }_{A}^{2}$ and ${\sigma }^{2}$) derived using the entire training set have been commonly used for calculating the heritability (using Eq. 17) which is usually used for assessing the performance of the GS model. However, using over-saturated markers along genome will overestimate the genetic variance (${\sigma }_{A}^{2}$), leading to exaggerated heritability (overfitting) in GS analysis. Here, we propose an objective evaluation of GS model by estimating the heritability through cross validation.

**Figure 1:**
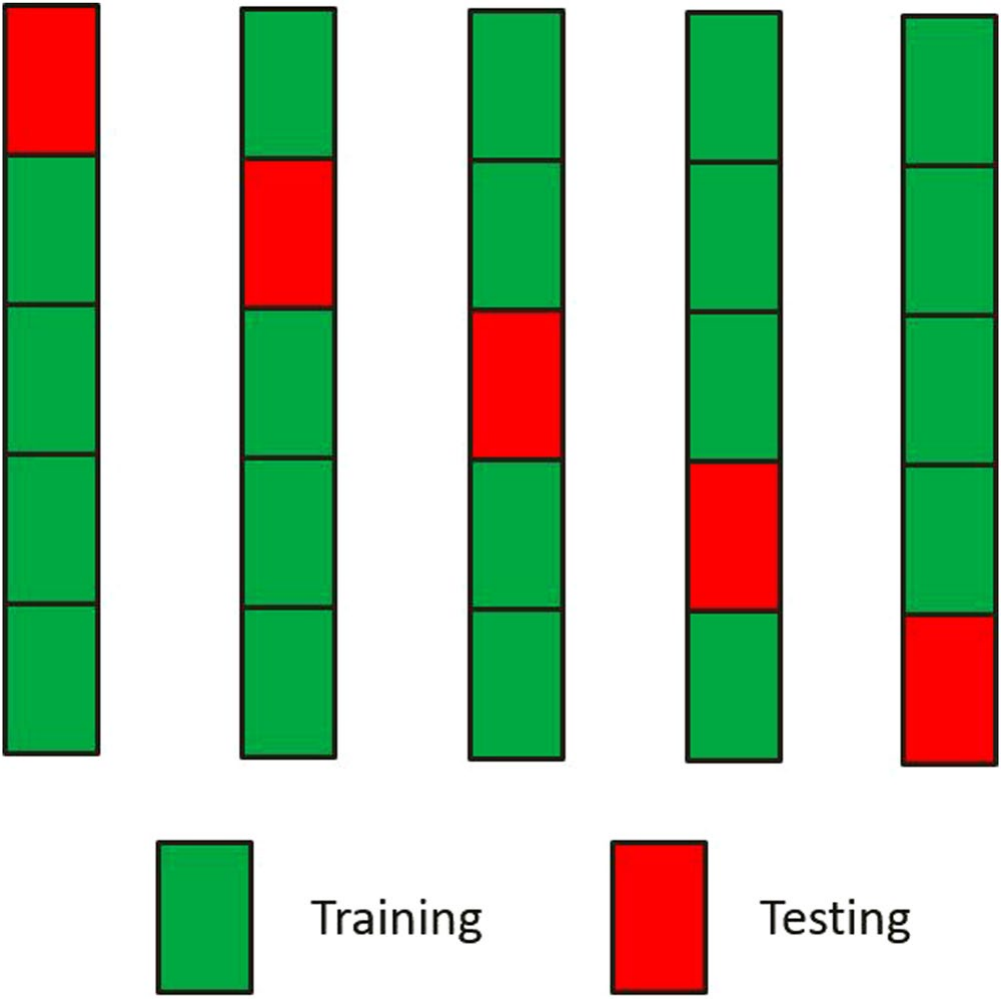
Demonstration of 5-fold cross validation.

Cross validation is often used to provide an objective assessment for the performance of a model^23^, and it has been used for MAS analysis and GS analysis to reduce the unwanted bias^20,21^. In cross validation, data is arbitrarily partitioned into two parts: training set and test set. The training set is used to estimate the model parameters (model development) and the test set is used for model evaluation regarding the predictability of the model. Thus, the test set does not contribute to model development at all; rather, the test set provides an objective evaluation on the performance of the model that is developed solely on the training set. In *k*-fold cross validation, data are randomly partitioned into *k* equal portions. Each time, *k*-1 portions are used to develop the model (calculate the model parameters); whereas, the remaining 1 portion is used for test. This process is repeated until each portion has been exactly used for once as test set. After *k*-fold cross validation, each subject has an observed phenotype and a predicted phenotype. The predicted phenotype is the value calculated when the subject is included in the test set during the cross validation. Figure 1 gives an example of 5-fold cross validation which is used in the current study. Rather than using the entire data to calculate genetic variance (${\sigma }_{A}^{2}$) and residual variance (*σ* ^2^) as in regular GS settings, we propose using the predicted genetic values (*via* cross validation) and the difference between the observed phenotype and the predicted genetic values to calculate the genetic variance and the residual variance, respectively, which are thereafter used to calculate the heritability (using Eq. 17). We will demonstrate through simulated studies that the heritability calculated using the predicted values through cross validation is equivalent to the predictability which is the squared correlation between the predicted phenotypes and the observed phenotypes (Eq. 25).

### Data set

The rice data used in the study includes 210 recombinant inbred lines (RILs), for each of which four traits [yield (YD), 1000 grain weight (GW), tiller number (TN), and grain number (GN)] have been replicated 4 times in different years and different locations^24^. High-density markers are used to infer recombination breakpoints^25^, facilitating construction of bins (1619 bins in the study) which are treated as new synthetic markers.

### SAS scripts for implementation of GS

The SAS scripts for implementation of GS using Mixed PROC is provided in below textbox. We first read in the dataset named ‘data’ that contains the phenotypic data (y) and dummy variable (X = 1) for individuals in the sample. The kinship matrix *K* and the identify matrix for residual (e) are specified in the general linear covariance structure for estimation of the variance components. The residual variance is fixed at 0.0001. Refer to online SAS User’s Guide for detailed instructions of using Mixed PROC (https://support.sas.com/documentation/). These SAS scripts need to be built in a MACRO to run a cross validation.
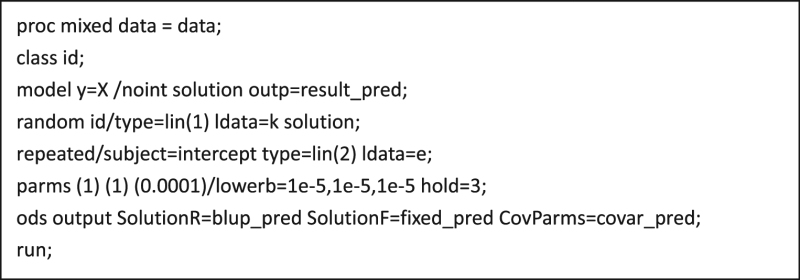


proc mixed data=data;

class id;

model y=X/noint solution outp=result_pred;

random id/type=lin(1) ldata=k solution;

repeated/subject=intercept type=lin(2) ldata=e;

parms (1) (1) (0.0001)/lowerb=1e-5,1e-5,1e-5 hold=3;

ods output SolutionR=blup_pred SolutionF=fixed_pred CovParms=covar_pred;

run;

## Results and Discussion

### Analysis of Rice Data

In the current study, genotype by environment (G × E) interaction was not considered; thus, for each trait, we treated the four observed phenotypic values for each RIL as four simple replicates. We first calculated the average of the 4 values of each trait for each RIL. Using SAS Mixed Procedure or R\GSMX, we first analyzed the averaged phenotype using the regular GS analysis (without cross validation). Note that the variances of genetics (${\sigma }_{A}^{2}$) and residuals (*σ* ^2^) are calculated using the entire data set. The results are presented in Table 1. We then applied the proposed algorithm to estimate the predictability and heritability for each trait using predicted genetic values through 10-fold cross validation (repeated 10 times for various data partitioning), which are also presented in Table 1.

**Table 1:** Results from the analysis of the rice data for 4 traits: yield (YD), 1000 grain weight (GW), tiller number (TN) and grain number (GN). The 10-fold cross validation has been repeated 10 times and the numbers in parentheses are the standard deviations of the averages.

GS Method	Trait	Variance		Heritability	Predictability
		Genetic	Residual		
Regular	YD	17.63	11.80	0.60	—
	GW	10.63	0.55	0.95	—
	TN	2.17	0.37	0.85	—
	GN	647.85	126.56	0.84	—
Cross validation	YD	17.63	11.8	0.19 (0.01)	0.18 (0.02)
	GW	10.63	0.55	0.75 (0.01)	0.75 (0.01)
	TN	2.17	0.37	0.52 (0.01)	0.52 (0.02)
	GN	647.85	126.56	0.40 (0.01)	0.40 (0.01)

The results showed that in non-cross-validation setting, the heritability appeared to be unrealistically larger than that shown in cross-validation setting. This is because, in non-cross-validation setting, a large number of neutral loci are used in regression analysis which overfitted the data and then overestimated genetic variances and the heritability. Whereas in cross-validation setting, the genetic variances and residual variance are calculated using predicted genetic values through cross validation, which provides a certain level of control for the potential overfitting in the training process. Note that, in cross-validation setting, the trait heritability calculated using the predicted genetic values is close to the trait predictability.

### Analysis of Simulated Data

**Figure 2:**
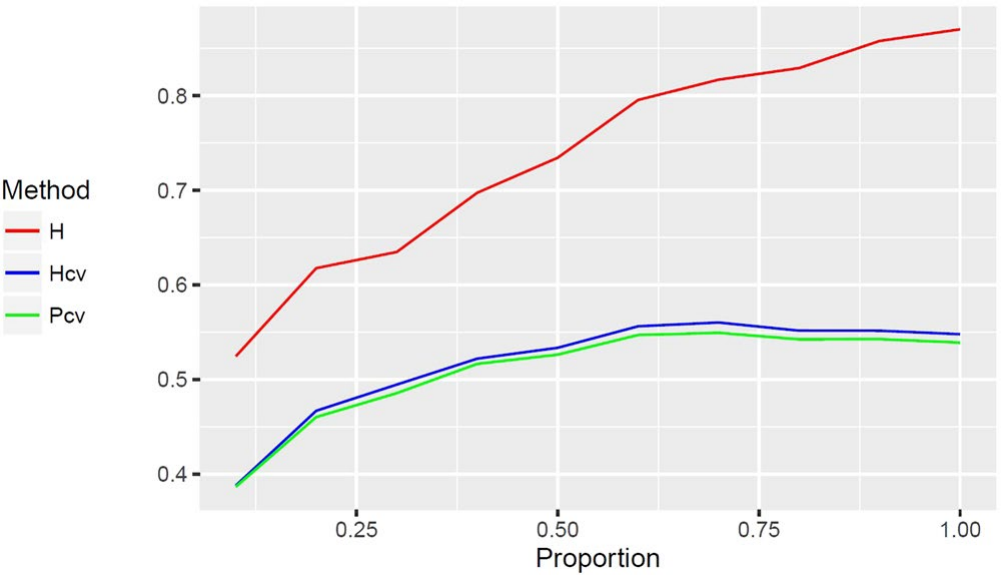
Analysis of simulated data with loci being continuously added to the GS analysis. X axis in each plot represents the percent of the sorted loci that have been included in the analysis. Y axis in each plot represents the achieved heritability or predictability with or without cross validation. H: heritability without cross validation; Hcv: heritability with cross validation; Pcv: predictability with cross validation.

In order to demonstrate that genetic variances are overestimated in the non-cross-validation setting (indicated in Table 1), we did the following simulated studies. We adopted the genotypes of the 1619 loci for each of the 210 RILs such that the natural genetic relationship between these RILs are preserved. For each of the 1619 loci, we simulated a genetic effect which is independently sampled from a normal distribution, *i*.*e*., ${\rm{N}}(0,\frac{1}{400})$. We only consider a single trait, for which the phenotypic values for each of the 210 RILs were calculated by multiplying the genotypes and the genetic effects plus a random error which is independently sampled from a normal distribution, *i*.*e*., N(0,1). Note that the ratio of the standard deviation of a genetic effect and the standard deviation of the residual was about 1/20 such that the overall heritability (accumulated from 1619 loci) was close to 50%. We calculated the correlation between the genotypes of each locus and the simulated phenotypic values; the 1619 loci were then sorted based on the strength of the association with the phenotype from the strongest association to the weakest association (the absolute value of Pearson’s correlation coefficient: min = 0.00026, median = 0.07707, max = 0.33090). The top 10% of the loci represent the most relevant QTLs (absolute correlation ranges from 0.20677 to 0.33090). We first analyzed the data only with the top 10% loci to calculate the heritability with or without cross validation and the predictability with cross validation (the initial values for each curves in Fig. 2). Then, we repeated the analysis each time with additional 10% loci in the sorted list being added to the data until all 1619 loci were eventually included in the analysis. The results are summarized in Fig. 2. The results show that if the top 30% most relevant loci (absolute correlation ranges from 0.12020 to 0.33090) have been included in the analysis, the heritability (blue curve) and predictability (green curve) calculated from cross-validation setting do not change very much when additional neutral loci are added to the data. On the contrary, with more and more neutral genes being added to the data, the heritability calculated in non-cross-validation setting continuously increased (red curve). This results supported our hypothesis that, without the control by the cross-validation in GS analysis, including irrelevant loci will overfit the data, and subsequently overestimate the genetic variance and eventually the heritability. If only the top 10% loci were used in the GS analysis, Hcv and Pcv could not reach maximum, which indicated that the model is not optimal at this point. This is because that many relevant but only weakly associated loci were not included yet, yielding an incomplete modeling. When most relevant loci were included in the GS analysis, Hcv and Pcv tend to be quite stable, suggesting that cross validation provides desirable control on overfitting due to the inclusion of neutral loci in the GS analysis. In general, the heritability calculated from non-cross-validation setting appeared to be much higher than those calculated from cross-validation setting, supporting the speculation of overfitting aforementioned. Moreover, the heritability calculated from cross-validation setting is very similar to the predictability that was calculated from the cross-validation setting (green and blue curves are close to each other in Fig. 2). An alternative approach to calculate Hcv is to use the ratio of the variance of the predicted genetic values (via cross validation) and the variance of the observed phenotypic variance.

We further did the following simulated study to demonstrate the deficiency of ANOVA analysis when compared with the GS analysis with cross validation. We also adopted the genotypes of the 1619 loci for each of the 210 RILs. Similarly, for each of the 1691 loci, we simulated a genetic effect from a normal distribution, *i*.*e*., N(0,*σ* ^2^), where $\sigma$ was chosen to be 1/10, 1/20, and 1/50, respectively. The phenotypic values for each of the 210 RILs were calculated with the same manner, *i*.*e*., by multiplying the genotypes and the genetic effects plus a random error which was independently sampled from a normal distribution, *i*.*e*., N(0,1). We chose different σ_g_:σ_e_ ratios in order to simulate scenarios with various levels of overall or accumulated heritability (ranging from 0.2 to 0.8). Note that these overall heritability is equivalent to the heritability that is calculated using GS model *without* cross validation. Therefore, the overall heritability only reflects the property of the entire training set; however, it does not indicate how well the genetic model developed from this training set would predict when it is applied to an independent set. This is the main point that we hope to address in the study. For each of the 210 RILs, we simulated 4 simple technical replicates (4 replicated measurements without additional block/replicate effect). We analyzed the data using three approaches: GS without cross-validation, GS with cross-validation, and ANOVA. Using GS analysis (either with or without cross-validation), we were able to analyze the 210 lines in each of the four replicated experiments separately, or analyze the average (a single value) of the four replicated measurements. When we analyzed the averaged phenotypes using GS analyses, the sample size appears to be 210; however, since the sample mean (sufficient statistic) is used for the GS analysis, the effective sample size for this analysis is actually 210 × 4 = 840. However, when we analyzed the 210 lines in each of the four replicated experiments separately, the effective sample size is only 210. The heritability without cross validation (H), the heritability with cross validation (Hcv), and the predictability with cross validation (Pcv) were calculated for each data analysis. In the ANOVA, all 840 phenotype values (210 lines × 4 replicates) were used to fit a linear regression model in R: Y ~ as.factor(line) + as.factor(replicate). The results from the three approaches are presented in Table 2.

**Table 2:** Analysis of simulated data with different levels of heritability. H: heritability; Hcv: heritability calculated through cross validation; Pcv: predictability calculated through cross validation. In this simulation, we generated 4 replicates for each of 210 individuals.

${{\rm{\sigma }}}_{g}/{\sigma }_{e}$		Replicate				Average	ANOVA
		1	2	3	4	Rep 1–4	Rep 1–4
	Sample Size	210	210	210	210	210	840
1/50	H	0.229	0.449	0.210	0.166	0.670	0.189
	Hcv	0.012	0.136	0.008	0.006	0.215	—
	Pcv	0.014	0.128	0.001	0.002	0.226	—
1/20	H	0.732	0.728	0.783	0.749	0.921	0.580
	Hcv	0.322	0.358	0.440	0.323	0.660	—
	Pcv	0.317	0.364	0.447	0.318	0.654	—
1/10	H	0.888	0.941	0.881	0.914	0.977	0.784
	Hcv	0.628	0.666	0.588	0.610	0.811	—
	Pcv	0.610	0.665	0.584	0.604	0.809	—

The results in Table 2 clearly show that (1) the trait predictability is equivalent to the trait heritability with cross validation; (2) the trait heritability calculated with non-cross-validation setting is consistently higher than those from cross-validation settings, indicating overfitting due to oversaturated markers in the analysis (proved in Fig. 2); (3) trait heritability and trait predictability substantially gained when the average of replicated phenotypes are used in GS analysis because the effective sample size becomes larger; (4) the trait heritability calculated from the ANOVA is different from the trait heritability calculated from the GS analysis through cross validation; (5) with the same effective sample size (840 in this simulated study), using the average trait value in GS analysis with cross validation enjoys higher heritability than using the ANOVA analysis.

In the ANOVA analysis, we no longer analyzed the variance based on the genotypes of the markers; rather, we analyzed the variance between groups (or RILs) which represent a new independent variable derived from the genotype data. This gives a good explanation to the aforementioned observation (4). In addition, another type of overfitting is possible if more groups than necessary are used in the ANOVA analysis. For example, samples with similar genotypes may be placed into different groups (or RILs). We calculated the pair-wise correlations of genotypes between the 210 RILs. It shows that the absolute correlation coefficient ranges from 0.0000016 to 0.9539, with 8 absolute correlation coefficients greater than 0.8. An ANOVA model with more than necessary groups/parameters certainly overfits the data. This is analogous to the situation where too many ‘leaves’ or ‘branches’ are used to fit data with a ‘tree’ classification model. Therefore, the heritability calculated using ANOVA with reorganized data is likely to be overestimated.

We further demonstrated the limitation of ANOVA when compared to the GS analyses using the following simulated study. Like the previous simulation, we simulated a genetic effect for each of the 1619 loci. The genetic effects were sampled independently from a normal distribution, *i*.*e*., ${\rm{N}}(0,\frac{1}{400})$. The phenotypic value for each of the 210 RILs was calculated by multiplying the genotype and the genetic effect plus a random error which was independently sampled from a normal distribution, *i*.*e*., N(0,1). The ratio of the standard deviation of a genetic effect and the standard deviation of the residual was about 1/20 such that the overall heritability in this simulation was about 50%. For each of the 210 RILs, we simulated different numbers of simple replicates, *i*.*e*., 10, 50, 100, 500, 1000, and 5000 replicated measurements. We first analyzed each dataset using ANOVA. The heritability did not change as the sample size increases (Haov in Table 3). We then averaged the replicated measurements for each RIL and analyzed the averaged phenotype using GS analysis with and without cross validation. The heritability calculated without cross validation (H), the heritability calculated with cross validation (Hcv), and the predictability with cross validation (Pcv) are listed in Table 3. Hcv and Pcv increased as the sample size grew, indicating that using larger samples boosts the statistical power for GS analysis. Whereas, increasing sample size does not help ANOVA at all. Moreover, ANOVA required replicated measurements to perform the analysis of variances by comparing the variance between groups and the variances within groups; increasing the number of replicates within groups does not help increase the heritability of the genetic model based on ANOVA. On the contrary, GS analyses do not require replicates; high level of statistical power for detecting the genetic effects may be gained by scrutinizing the genome-wide high density markers. From Table 3, it is obvious to see the overfitting due to the inclusion of neutral loci if cross validation is not applied in GS analysis (H). Also, we have proved again that the heritability (Hcv) is equivalent to predictability (Pcv) when cross validation is applied to GS analyses.

**Table 3:** Analysis of the simulated data with different numbers of replicates. Haov: heritability calculated using ANOVA; H: heritability; Hcv: heritability calculated through cross validation; Pcv: predictability calculated through cross validation.

Number of Replicates	Haov	H	Hcv	Pcv
10	0.5440	0.9707	0.8068	0.8082
50	0.5393	0.9981	0.8756	0.8774
100	0.5324	0.9951	0.8860	0.8894
500	0.5412	0.9999	0.8999	0.9038
1000	0.5424	0.9999	0.9073	0.9099
5000	0.5406	1.0000	0.9126	0.9162
